# Upper holocene tephro-chronostratigraphy of Irazú Volcano, Costa Rica

**DOI:** 10.1038/s41598-024-57962-7

**Published:** 2024-03-26

**Authors:** Daniela Campos-Durán, Guillermo E. Alvarado, Joan Martí

**Affiliations:** 1 Escuela de Ciencias Geográficas, Universidad Nacional de Costa Rica, Heredia, Costa Rica; 2 Geociencias, Instituto Costarricense de Electricidad (ICE), San José, Costa Rica; 3 Department of Geosciences, IDAEA, CSIC, Barcelona, Spain

**Keywords:** Irazú volcano, Upper Holocene, Radiometric dating, Tephra deposits, Tephro-chronostatigraphy, Volcanic hazards, Eruptive recurrence, Natural hazards, Solid Earth sciences

## Abstract

Irazú is one of the largest and most active volcanoes in Costa Rica. We present the tephro-chronostratigraphy of the last 2.6 ka of the Irazú volcano based on detailed field work and C^14^ radiometric dating, as well as a revision of the geological and historical records. In the stratigraphic record we identified at least 30 tephra units. Eight of them corresponding to the historical period (i.e., after 1700 A.D.), separated by repose periods of different durations. The distribution of the deposits, the volcanic morphologies (craters and pyroclastic cones) and the radiometric ages indicate that most of this recent eruptive activity has occurred from the summit of Irazú along an E–W fissure (~ 4 km long). Toward the west of the summit, near the Sapper hill may be the source of the oldest eruptions at 200 A.D., while the La Laguna cone, located to the east of the summit, could have formed around 1540 A.D., and Main Crater to the west could have formed around sixteenth–seventeenth century. Since then, the historical eruptions (i.e., 1723–1724, 1917–1921, 1924, 1928, 1930, 1933, 1939–1940 and 1963–1965) have been sourced from this crater, but not all of them are registered in the stratigraphy. The eruption frequency of Irazú during this period ranges from 23 to 100 years, with a major event about every 80 years. Irazu’s eruptions have been mainly phreatomagmatic and Strombolian, including some phreatic explosions. We present a detailed tephro-chronostratigraphy that will help to building temporal analysis for hazard assessment and risk management plans to face future eruptions at Irazú.

## Introduction

The characterization of the eruption record of a volcano is is fundamental to establish its eruption frequency and to determine its eruption dynamics (e.g.,^1,2^). This is necessary to conduct long term hazard assessment and to identify the main eruptive scenarios and their associated potential impacts^3–5^. Field volcanic stratigraphy and adequate radiometric dating methods are necessary to build a solid chronological framework of eruptive events. In this sense, the tephrochronology is a unique stratigraphic method for linking, dating, and synchronizing geological events^6,7^. The radiocarbon dating is widely used to date Holocene and Late Pleistocene volcanic events and is helpful for archaeological and other related studies (e.g.,^8,9^). Successful examples of tephrostratigraphic studies have been carried out in different volcanic areas (e.g., Iceland^10,11^; Jan Mayen^12^; New Zeland^13^; Faroe Islands^14^; Japan^15^; Central Mediterranean^16^; Kamkatchka^17^, thus indicating the importance need of such investigations to constraint Pleistocene-to-Holocene volcanic evolution and as necessary step in hazard assessment. Unfortunately, despite these efforts, it is not always possible to fully reconstruct the geological record of a volcano, or of a selected part of it, particularly when we try to go beyond its historical period, as some deposits may have been partially or totally eroded or buried by more recent deposits, or, as it is in our case, covered by dense grass and forest, which results in limited outcrops and a poor preservation of tephra.

**Figure 1:**
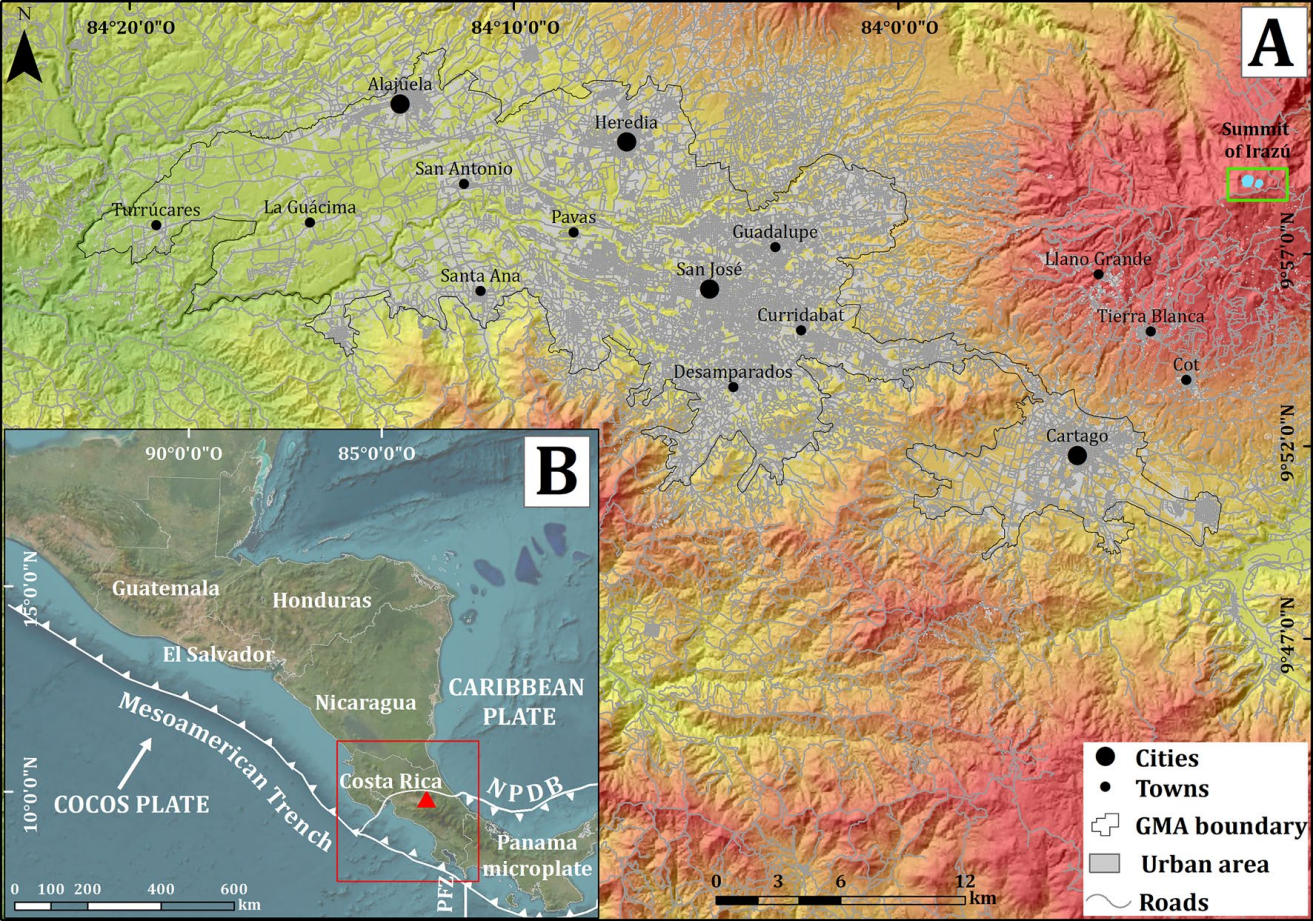
(**A**) Location of Irazú volcano in the Central Volcanic Cordillera of Costa Rica and its position respect to Great Metropolitan Area (GMA), where the cities of San José, Alajuela, Cartago, and Heredia are located. See reference of the DEM used in the Methodology section (**B**) Geodynamic setting of Costa Rica. NPDB: North Panama Deformed Belt, PFZ: Panama Fracture Zone. Satellite image modified from Google Earth.

**Figure 2:**
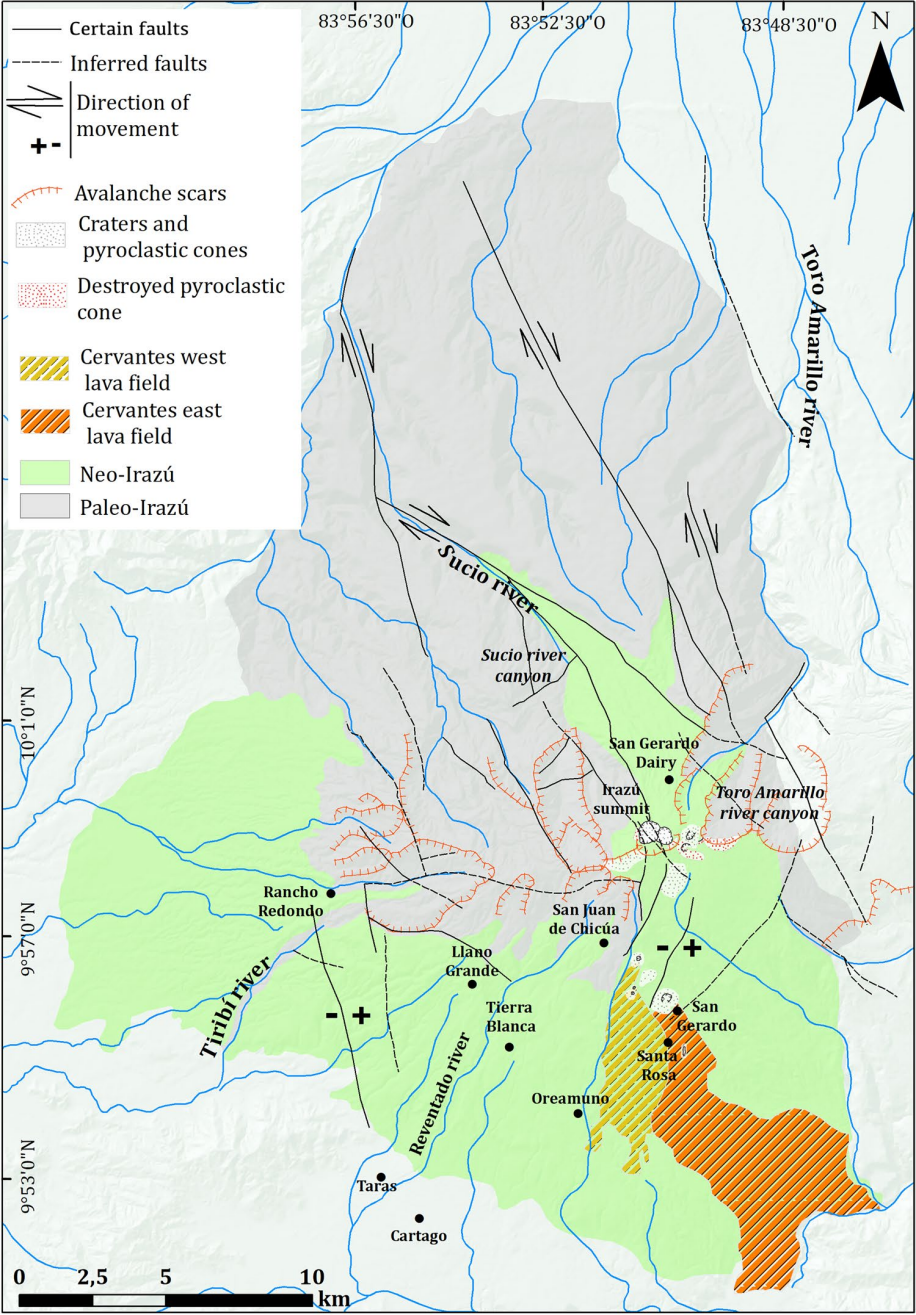
Main pyroclastic cones on the southern flank of the summit and the main avalanche scarps both north and south of the summit. Also, it shows the Irazú constructive units (proto-Irazú and Paleo-Irazú) and the two large lava fields Cervantes West and Cervantes East (57 ka and 17 ka, respectively) and finally the main faults.

The Irazú volcano is one of the most studied volcanoes in Costa Rica. Several investigations have provided valuable information about its geology, petrology, stratigraphy, geochronology, tephrostratigraphy, and chemistry^18–24^. However, its eruption history is still poorly known and there are scarce data to characterize it. To date 19 tephrostratigraphic units from the last 2.6 ka, located at intermediate distances (up to 6 km) from the summit of the Irazú volcano were proposed by^20^. However, this record could be significantly improved considering the eruption frequency and copious tephra deposition during historical, and likely pre-historical eruptions. Between 1723 and 1965 the Irazú volcano has shown several eruptive episodes of different intensity and duration^25–30^. One of the most important eruptive episodes occurred between 1963 and 1965 and reaching a maximum Volcanic Explosivity Index (VEI) of 3 according the distribution thickness of fallout deposits and maximum high reached by the eruption column^19,30^. Ash fall and the lahars caused damage to infrastructure (houses, bridges, factories, and roads) and significant economic loss (mainly in agriculture and livestock) in the Great Metropolitan Area (GMA) mainly in the cities of San José and Cartago (see Fig. 1A), two of the main urban areas of the country, as well as > 20 fatalities due to the lahars that occurred on December 9–10, 1963 in the Reventado river sub-basin, in sites comprised between Tierra Blanca, Taras and Cartago^19,30,31^ (Fig. 2). Previous studies indicate that Irazú can be considered as a high-threat volcano, due to its potential associated hazards and the degree of exposure of neighboring areas^19^ for which establishing a precise chronology and characterization of its last eruptive events is central to conduct further long-term hazard assessment.

This paper provides a detailed reconstruction of the tephro-chronostratigraphy of the Irazú volcano for the Upper Holocene. Based on the identification of its field volcanic stratigraphy supported by new 11 radiocarbon dating (^14^C). In addition, we also correlate these volcanic deposits with their possible vents, which help to infer the most probable source region for future eruptions.

## Geological setting

Irazú is an andesitic shield volcano located at the Central America Volcanic Arc, to the east of the capital city of San José (Fig. 1A,B), built on an old Pliocene volcanic basement, which reaches an altitude of 3427 m a.s.l. The volcanic edifice covers an area of approximately 700 km^2^, with a volume of 359 km^3^. Volcanic activity at the arc results from the subduction of the Cocos plate beneath the Panama microplate (Fig. 1B). The depth of the subducted Cocos plate, below the peak of Irazú, appears to be about 110 km^32^. The Moho has been delimited seismically between a depth of 35 and 45 km^33–35^.

Irazú shows a broad summit central complex with two main craters (Main and Diego de la Haya craters), the first one being currently occupied by a small lake. There are also several satellite cones on its South flank and E-W summit, as well as several landslide scars affecting most of its flanks (Fig. 2)^19^. The formation of Irazú volcano dates back to less than a million years; the ^40^Ar/^39^Ar radiometric dating available so far groups the eruptive products into three significant age units: a) the proto-Irazú at 0.85 Ma (San Jerónimo Ignimbrite), b) the paleo-Irazú between 0.6 and 0.25 Ma, which is consistent with eroded volcanic structures and lava platforms, and c) effusive and explosive products younger than 0.2 Ma, culminating in the two large lava fields Cervantes West (57 ka) and Cervantes East (17 ka) (Fig. 2). The Cabeza de Vaca (0.594 ± 0.016 Ma) and Finca Liebres (251 ± 4 ka) extinct volcanoes are also part of the complex evolution of paleo-Irazú^21,23,24,36^.

The structural control of Irazú is expressed by several faults with NE-SW, E-W, and NW–SE orientations that are visible on its flanks (Fig. 2)^37–39^, which have also controlled the location of the craters and eruptive vents^40^. Another aspect to consider are the thermal anomalies detected by infrared images as well as the thermal sources and fumaroles identified in the field^41–43^, which are likely aligned in the same predominant structural patterns.

**Figure 3:**
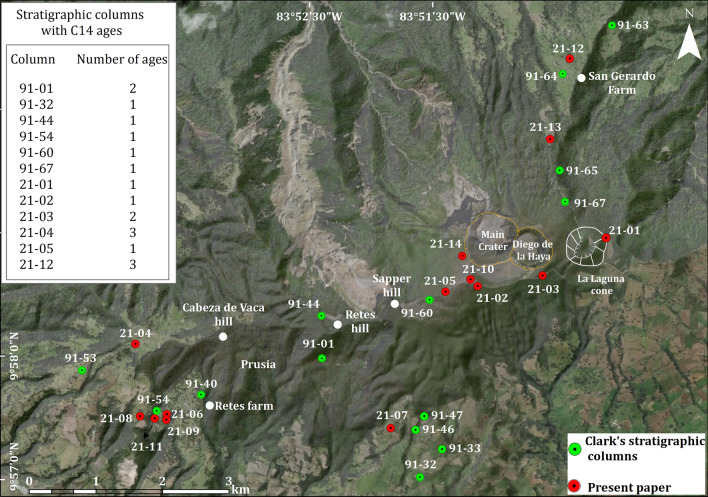
Location of the previous^20^ and new stratigraphic columns on the summit and SW and NE flanks of Irazú volcano.

Volcanic activity at Irazú has been mostly characterized by lava flows, Strombolian and Vulcanian eruptions. Some of its slopes have been deeply modified by the occurrence of sector collapses that have left several horseshoe-shaped structures still well preserved. On present edifice several craters and parasitic cones are recognizable on its summit and flanks. The summit consists of an E-W oriented ridge, (Fig. 2). The uppermost part is occupied by two main craters: Principal and Diego de la Haya craters (Figs. 2 and 3). The most recent and historical activity has occurred from the westernmost crater (Main Crater)^19^. The earliest reported eruption of Irazú in 1723 produced violent Strombolian fountains followed by phreatomagmatic explosions. All subsequent verifiable eruptive events took place during the last century: 1917–1921, 1924, 1928, 1930, 1933, 1939–1940, and 1963–1965 (Table 1 for more details)^21^. The last event, in 1963–65^30^ generated proximal hydrothermally altered breccias, scoria-fallout, dilute pyroclastic density currents (PDCd) and fine ashes which were deposited downwind for several tens of kilometers to the W-SW, causing a considerable impact on area of 3000 km^2^. Subsequent severe rainfall remobilized part of this ash fall deposited on flanks of the volcano and triggered over 90 flash floods and lahars that affected the Cartago area causing 20 fatalities and destroying 400 buildings^43^.

**Table 1:** Characterization of the volcanic activity of Irazú in historical time.

Year	Duration (months)	Type of eruption	Maximum height of the eruptive column above crater level (km)	Type of deposit	VEI max	Effects
1723–1724	≥ 12	Strombolian, phreatomagmatic and phreatic	> 2	Ash, scoria lapilli, PCDd and bombs	3	Damage to one house
1917–1921	≥ 44	Phreatomagmatic	~ 5.5	Ash, ballistic and PCDd	2	No record of damage
1924	~ 2	Phreatomagmatic	N/D	–	~ 1	
1928–1930	~ 6.5	Phreatomagmatic	> 2	–	2	
1933	4	Phreatomagmatic	~ 5.5?	–	2	
1939–1940	9	Phreatomagmatic	~ 4.5	Ash and PCDd	2	Damage to plantations and livestock
1963–1965	30	Strombolian, phreatomagmatic and phreatic	8	Ash, lapilli, scoria, PDCd and ballistic	3	Damage to plantations, livestock, and infrastructure

Despite their low volcanic explosivity index (VEI < 3)^19^, Irazú’s historical eruptions have caused fatalities, serious agricultural losses, and damage to infrastructure, mostly by lahars and persistent ash fall on the GMA (Fig. 1A), the most populated area of Costa Rica^44^. Primary pyroclastic products have been generated during Strombolian and Vulcanian eruptions, but phreatic explosions from the main Crater have been also frequent, producing widespread fine ash deposits. Sector collapses affecting part of the Irazú flanks have been also frequent at Irazú, thus also representing a main hazard for the most proximal areas. Tectonic and volcano-tectonic earthquakes swarms have been frequent in the Irazú area, reaching magnitudes up to 5.9 Ms^37^.

## Materials and methods

Volcanic terrains in tropical areas are heavily affected by weathering and vegetation growth, which covers most of them in thick soils. Irazú volcano is not an exception and most of it is covered by a dense forest, mainly toward the N flank where a dense rain forest exists. In particular, Irazú field work in 2020 to 2022 was hampered by: (a) the conditions of the few existing access roads, sometimes impracticable even with a four wheel drive car, (b) the outcrops on the road were usually covered by vegetation, so most of them had to be cleaned with a shovel, thus limiting its lateral observation, (c) on some occasions the rainy conditions made it difficult to clean the outcrops and to describe them, and (d) most of the outcrops visited where in private properties, so we had to obtain permits to access them. Nevertheless, fourteen detailed stratigraphic columns were established in the proximal areas of Irazú (< 6 km), where the deposits are exposed and accessible, mainly on its NE and SW flanks, while most of other areas are highly urbanized or completely covered by vegetation (Fig. 3).

Field data were transferred to a digital database built on a DEM whose characteristics are: DEM obtained from aerial photographs in 2005–2007, at a scale of 1:25,000, reference system: EPSG: 5367, projection: Transversal de Mercator for Costa Rica (CRTM05), Ellipsoid WGS84, Datum CR05, Credit: Department of Geography—IGN (https://www.snitcr.go.cr/ico_servicios_ogc). Correlation between outcrops was established using volcanic-stratigraphic criteria (e.g.,^7^) and comparison of the lithological and sedimentological characteristics of the deposits. Thickness measurements, as well as lithological characteristics (see Tables 2 and 4) were obtained for all volcanic deposits from these stratigraphic sections. These new stratigraphic sections were combined with the existing ones from Clark^20^, which we revised in the field (Fig. 3 for location). Also, we included four fallout deposit units (González, Dóndoli, Tristán, and Alfaro) previously identified by^19^ and^21^. Another important and distinctive tephra layer from the Turrialba volcano (10 km NE of Irazú), dated by^45^ to 2010 ± 60 yr B.P., and which is identifiable on the NE and SW flanks of Irazú, was used as a marker horizon in that area to refine the stratigraphic correlation of the overlying deposits from the Irazú volcano.

**Table 2:** General lithological and sedimentological characteristics of studied deposits.

Type of deposit	Lithology and composition	Sedimentary structures	Location
Proximal fallout	Compositionally and texturally discrete stratigraphic packets of coarse to medium size ash, well sorted and layered deposits, mantle the topography, including non-gradedd, juvenile escoriaceous bombs and scoria lapilli layers, and impact bombs and blocks. The dominant color is dark to light gray, to brown color. Some deposits are rich in hydrothermally altered ash and lithic fragments, with variable colored (orange, red, with, brown). Petrographically, the rocks are basaltic to andesite. < 8% of non-juvenile lithic fragments	Symmetric to asymmetric grading (normal or reverse to normal), but also non-graded and coarse-grained character in layers in some deposits implying short transport distance, and the orientation of asymmetric ballistic impact sags showing the source	Summit and near vents
Distal fallout	Widespread fine to medium size ash and lapilli deposits, well sorted, massive to layered sequence. The dominant color is dark to light gray, to brown color, with very thin layers of yellow to strong orange or pale red color. Compositoinally, the juvenile components are basaltic andesite to andesite. < 3% of non-juvenile lithic fragments	Massive, laminated or stratified parallel layering and mantling the topography, well sorted and normal gradding	SW flank NE flank
Dilute Pyroclastic Density Currents (PDCd)	Fine grain, thinly laminated, well sorted, fine to medium ash beds deposits, including massive layers. The dominant color is dark to light gray, orange, or brown color. The juvenile components are basaltic andesite to andesite in composition. 25–45% of non-juvenile lithic fragments	Flat upper surface and an irregular lower surface with low-angle, cross to dune lamination (sand wave), pinch and swell structure to truncation structures, erosive channel filled. Passage between cohesive to non-cohesive deposits is sharp but normally without any erosional surface. Some shows soft sediment deformation (contorned stratification) and slide blocks. Plasted ash deposits against trees and vertical walls	Summit NE flank
Agglomerate	Massive bomb deposits with juvenile block and scoria lapilli layers, and rare hydrothermally altered lithic deposits. Petrographically, the deposits show characteristics of magma mingling, present as spatter fragments and scorias with a black and white banded texture, which in composition are basaltic andesite and dacite, respectively. Up to 5% of non-juvenile lithic fragments	Distinctive poorly structureless horizons of welded bombs, with little or no internal layering	Summit and near vents
Agglutinate	Massively welded, deformed bombs and spatter fragments, crudely layered with angular to subangular glassy clast to highly vesicular bombs and lapilli, and rare hydrothermal altered lithics. The deposits are of basaltic to andesite composition. < 3% of non-juvenile lithic fragments	Distinctive structureless horizons of welded and flattened bombs, with little or no internal layering	Summit and near vents

Therefore, the tephrostratigraphy of uppermost part of Irazú volcano was established combining the new stratigraphy, all the available radiocarbon data, and the existing historical records from which there is a geological correspondence (i.e., presence of tephra units in the field). The stratigraphic order from base to top and the name of the different stratigraphic units, was elaborated considering the original stratigraphy nomenclature from^20^, named, in an aging order, with letters A (historical tephras) and from B to S (pre-historical tephras). In order to preserve this nomenclature when new layers were added, we used numbers (e.g., A1, A2, C1, C2, etc.) following the main unit identified by^20^. Some of these former stratigraphic units were revised and adapted to the new stratigraphy when their position disagreed with the new data. The new and former radiocarbon dates (18 in total) were associated with the corresponding stratigraphic units (Table 3). The result obtained is shown in Table 4, where 30 tephra units, names from P to A4 in stratigraphic order, are identified in the last 2.6 ka of the Irazú volcanological history.

**Table 3:** ^14^C ages of the Irazú volcano (B. P.: before present, the present is the year 1950 A.D.; B. C.: before Christ; A. D.: Anno Domini, similar to after Christ).

Stratigraphic section	Coordinates	Site Description	Conventional age (yr B.P.)	Calibrated age using the Northern Hemisphere curve (IntCal20)	Source
21–12-03	10° 00′ 23″ N 83° 50′ 23″ W	~ 250 m E of the San Gerardo farm	0 ± 30	1876–1916 A.D	This work
91–01-10	9° 57′ 58″ N 83° 52′ 28″ W	Prusia, ~ 440 m SW of Retes hill, El Roble Trail	315 ± 20	1521–1577 A.D	^20,22^
21–01	9° 58′ 56″ N 83° 50′ 05″ W	~ ~ 400 m N of the main entrance of Irazú Volcano National Park. La Laguna cone	330 ± 30	1549–1598 A.D	This work
21–12-02	10° 00′ 23″ N 83° 50′ 23″ W	250 m E of the San Gerardo farm	410 ± 30	1432–1520 A.D	This work
21–02	9° 58′ 32″ N 83° 51′ 08″ W	~ 200 m SE of Irazú Volcano National Park Viewpoint	510 ± 30	1409–1435 A.D	This work
21–12-01	10° 00′ 23″ N 83° 50′ 23″ W	250 m E of the San Gerardo farm	730 ± 30	1254–1302 A.D	This work
91–44-09	9° 58′ 18″ N 83° 52′ 28″ W	Top of Retes hill	920 ± 60	1040–1175 A.D	^20,22^
21–03-01	9° 58′ 38″ N 83° 50′ 36″ W	50 m W of the new Irazú Volcano National Park payment station, on the way to the crater	1110 ± 30	895–990 A.D	This work
91–67-05	9° 59′ 14″ N 83° 50′ 30″ W	~ 800 m N of the La Laguna cone, from Route 219	1230 ± 70	780–883 A.D	^20,22^
21–03-02	9° 58′ 38″ N 83° 50′ 36″ W	50 m W of the new Irazú Volcano National Park payment station, on the way to the crater	1300 ± 30	740–773 A.D	This work
91–01-05	9° 57′ 58″ N 83° 52′ 28″ W	Prusia, ~ 440 m SW of Retes hill, El Roble Trail	1325 ± 35	657–687 A.D	^20,22^
21–04-01	9° 58′ 04″ N 83° 53′ 54″ W	Road cut at Cabeza de Vaca farm	1440 ± 30	604–643 A.D	This work
21–04-02	9° 58′ 04″ N 83° 53′ 54″ W	Road cut at Cabeza de Vaca farm	1610 ± 30	496–534 A.D	This work
21–04-03	9° 58′ 04″ N 83° 53′ 54″ W	Road cut at Cabeza de Vaca farm	1620 ± 30	415–533 A. D	This work
91–54-02	9° 57′ 29″ N 83° 53′ 51″ W	SW boundary of the Irazú Volcano National Park, near Retes hill	1600 ± 180	321–611 A.D	^20,22^
21–05-02	9° 58′ 30″ N 83° 51′ 23″ W	~ 400 m W of the Irazú Volcano National Park viewpoint. Carcava, to the right, after the ICE tower	1850 ± 30	158–236 A. D	This work
T-109–7	10° 0′ 24″ N 83° 50′ 23″ W	350 m E San Gerardo dairy	2010 ± 60	53 B.C.-81 A.D	^45^
91–32-01	9° 57′ 00″ N 83° 51′ 40″ W	Route 219, 400 m NE from San Juan de Chicuá	2530 ± 170	807–451 B.C	^20^

B. P., before present (the present is the year 1950 A.D.); B. C., before Christ; A. D., *Anno Domini* (similar to after Christ).

**Table 4:** Upper Holocene tephrochronology of the Irazú volcano (stratigraphic units are indicated from the youngest to the oldest), PS: paleosol; Erosive contact: EC.

Unit	Description and interpretation, based on previous studies ^20,22^	Conventional age ^14^C (B.P.)	Age*	Outcrop	References
A4	Coarse to medium gray ash and lapilli. Phreatomagmatic and stratified andesitic strombolian deposits (fallout and PDCd)		1963–1965 A.D	91–01, 91–44, 21–03, 21–14, 21–09, 21–06, 91–40	This work
EC					
A3	Thin levels of medium-fine ash with yellow to red coloration. Stratified phreatomagmatic deposits (fallout and PCDd)		1939–1940 A.D	21–14	This work
EC					
A2	Thin levels of medium-fine ash with yellow to red coloration. Phreatomagmatic deposits (fallout, ballistic and PCDd)		1917–1921 A.D	21–14	This work
PS	Brown paleosol				
A1	Black scoria with occasional pumice and coarse black ash. It presents thick phreatomagmatic breccia deposits that are mainly recognizable at the summit of Irazú. Phreatomagmatic and stratified andesitic strombolian deposits (fallout and dilute PDCd)		1723–1724 A.D	91–54, 91–01, 91–44, 21–03, 21–05	This work
PS	Brown paleosol				
Tencha	Hydrothermalized blocks of different sizes and hydrothermalized ash matrix). Overbank avalanche deposits		~ 1900 A.D	91–63, 91–64, 91–65, 91–67, 21–12, 21–13	^20,22^
PS	Paleosol with charcoal	**0 ± 30**	1876–1916 A.D		
B2	Fine to coarse ash, lapilli, and juvenile fragments with abundant lithic fragments of the same sizes and of different lithologies. Phreatic deposits (fallout)		~ 1561 A.D	21–01	This work
PS	Brown paleosol with ash and organic material	**330 ± 30**	1549–1598 A.D		
B1	Scoria (lapilli and bombs), non-juvenile blocks, orange ash, wedging. Bombs and blocks (La Laguna cone). Strombolian deposit (fallout)		~ 1540 A.D	21–01	This work
PS	Brown paleosol rich in altered ashes and charcoal	**315 ± 20**	1521–1577 A.D		
C4	Brown ash, towards the top, there is a scoriaceous level with hydrothermalized lithics and towards the base blocks. Phreatic deposit (fallout)		~ 1500 A.D	21–02	This work
PS	Brown paleosol rich in ash and altered tephra				
C3	Gray ash, coarse grain with wedging. Stombolian deposit (fallout)		~ 1460 A.D	21–02	This work
PS	Discontinuous brown to orange paleosol	**410 ± 30**	1432–1520 A.D		
C2	Orange to brown lapilli, poor selection. Phreatomagmatic deposit (fallout)		~ 1420 A.D	21–02	This work
PS	Paleosol	**510 ± 30**	1409–1434 A.D		
C1	Lapilli scoriaceous/ash, with scoriaceous bombs. The proximal area corresponds to a thick level of scoria. Strombolian deposit (fallout)		~ 1300 A.D	21–02, 21–10	This work
PS	Brown paleosol	**730 ± 30**	1254–1302 A.D		
PS	Brown paleosol	**920 ± 60**	1040–1175 A.D		
D4	Fine to a very fine ash, wavy to parallel, with pinkish layers. Towards the top lapilli with red clay, it is poorly sorted. Phreatomagmatic deposit (fallout)		~ 1000 A.D	21–03	This work
PS	Brown paleosol with charcoal and organic material	**1110 ± 30**	895–990 A.D		
D3	Coarse-grained brown ash, with lapilli and blocks. Predominantly phreatic deposit (fallout)		~ 850 A.D	21–03	This work
PS	Brown paleosol with charcoal and pre-Columbian pottery	**1230 ± 70**	780–883 A.D		
D2 (R Unit)	Lapilli (white and orange clasts) and intermediate coarse-grained brown to orange ash. Predominantly phreatic deposit (fallout)		~ 800 A.D	91–63, 91–64, 91–65, 91–67, 21–03	This work, ^20,22^
PS	Brown paleosol with charcoal				
D1	Coarse-grained, dark gray ash, stratified with fine lapilli. Stombolian deposit (fallout)		~ 700 A.D	91–54, 91–01, 91–44, 21–03, 21–06, 21–09, 21–08	Thjis work
PS	Brown paleosol with organic material	**1300 ± 30**	740–773 A.D		
PS	Paleosol with charcoal	**1325 ± 35**	657–687 A.D		
PS	Brown Paleosol	**1440 ± 30**	604–643 A.D		
E5	Fine to medium-grained ash, laminated with small orange layers. Strombolian deposit (fallout)		~ 540 A.D	21–04	This work
PS	Brown paleosol with hydrothermalized levels	**1610 ± 30**	496–534 A.D		
E4	Fine to medium-grained gray ash. Strombolian deposit (fallout)		~ 500 A.D	21–04	This work
PS	Brown to gray paleosol with charcoal fragments				
E3	Fine to medium-grained gray ash. Strombolian deposit (fallout)		~ 470 A.D	21–04	^20,22^
PS	Organic Paleosol				
E2	Fine to medium-grained gray ash and white lapilli. Strombolian deposit (fallout)		~ 460 A.D	21–04	^20,22^
PS	Brown paleosol				
E1	Medium-grain dark gray ash. Strombolian deposit (fallout)		~ 450 A.D	21–04	^20,22^
PS	Dark paleosol with organic matter and charcoal	**1620 ± 30**	415–533 A.D		
PS	Paleosol with charcoal	**1600 ± 180**	321–611 A.D		
F	Very fine ash, to the base orange, wavy, lenticular. Phreatomagmatic deposit (fallout)		~ 400 A.D	91–40, 91–53	^20,22^
PS	Brown Paleosol				
G	Fine to coarse-grained gray to orange ash with occasional weathered lapilli. Phreatomagmatic deposit (fallout)		~ 350 A.D	91–33, 91–47	^20,22^
PS	Brown paleosol, occasionally rich in clay				
H	Fine-grained gray ash, bioturbated. Strombolian deposit (fallout)		~ 300 A.D	91–40	^20,22^
PS	Brown paleosol with altered pumice, occasionally with charcoal				
I	Gray ash, medium to coarse grain, occasionally with accretionary lapilli and dispersed pumice. Phreatomagmatic deposit (fallout)		~ 250 A.D	91–40, 91–53, 91–54	^20,22^
PS	Brown paleosol rich in ash				
J	A distal layer of very fine to coarse gray ash with lenticular levels. In the proximal facies, it appears to be represented by an agglutinated. Strombolian deposit (fallout and agglutinate)		~ 200 A.D	21–05, 91–40	This work, ^20,22^
PS	Brown paleosol with charcoal	**1850 ± 30**	120–248 A.D		
K	Very fine-grained orange ash. Phreatomagmatic deposit (fallout)		~ 100 A.D	91–46, 91–47	^20,22^
PS	Charcoal fragment on the El Retiro layer (Turrialba Unit 4)	**2010 ± 60**	53 B. C. – 81 A.D		^45^
4	Subplinian layer of the Turrialba volcano		~ 25 A.D	91–54, 91–64, 91–63, 21–04, 21–07, 21–12, 21–13	
PS	Brown soil, rich in ashes and charcoal				
L	Medium to fine-grained gray ash with some discontinuous stratified horizons. Strombolian deposit (fallout)		~ 100 B. C	91–40, 91–53, 91–54	^20,22^
PS	Brown paleosol rich in ash				
M	Coarse grained gray ash with meteorized pumices. Phreatomagmatic deposit (fallout)		~ 200 B. C	91–44, 91–47	^20,22^
PS	Brown paleosol occasionally with dispersed pumice and orange ashes and charcoal				
N	Gray ash, medium to coarse grain and vesicular and scoriaceous lapilli (rare) with poor selection; accretionary lapilli. Phreatomagmatic deposit (fallout)		~ 300 B. C	91–32, 91–40, 91–46, 91–53, 91–54	^20,22^
PS	Brown to orange paleosol, rich in clay and charcoal	**2530 ± 170**	1048–340 B. C		
O	Very fine gray ash layer with occasional stratification. Strombolian deposit (fallout)		~ 800 B. C	91–53	^20,22^
PS	Brown paleosol rich in ash				
P	Gray ash, very fine to coarse grain, with orange and pinkish levels with discontinuous wavy layers. Phreatomagmatic deposit (fallout)		~ 900 B. C	91–54, 91–40	^20,22^
PS	Brown to black paleosol with charcoal		~ 1000 B. C		

Eleven samples from six outcrops (Fig. 3) corresponding to paleosoils underlying or overlying primarily pyroclastic deposits (fallout or pyroclastic density current (PDC), deposits) that contain sufficient organic matter were sampled for radiocarbon dating. The outcrops were carefully cleaned and then the material to be dated was taken, without touching it, from the soil matrix. Radiometric dating was carried out at Beta Analytic Laboratories (USA) using the accelerator mass spectrometry (AMS) dating. The Beta Analytic's BetaCal 4.2 calibration program, used the international IntCal20 database.

The Conventional Radiocarbon Ages were all corrected for total fractionation effects and where applicable, calibration was performed using probability method of^46^ and calibration database INTCAL20^47^. The results obtained are accredited to ISO/IEC 17025:2017 Testing Accreditation PJLA #59423 standards and all chemistry was performed here in the Beta Analytic Radiocarbon Dating Laboratory and counted in their own accelerators. Conventional Radiocarbon Ages and sigmas were rounded to the nearest 10 years per the conventions of the 1977 International Radiocarbon Conference. When counting statistics produced sigmas lower than + /− 30 years, a conservative + /− 30 BP was cited for the result. The reported d13C values were measured separately in an IRMS (isotope ratio mass spectrometer). A more detailed explanation of the analytical procedure is available at the company's website (https://www.radiocarbon.com/espanol/datacion-laboratorio.html). The new dates were combined with the existing ones^20,22^, giving a total of 18 radiometric ages for paleosoils intercalated with the Irazú tephras for the last 2600 years. All ages were calibrated with the Calib Rev 8.1.0 calibration program (http://calib.org/calib/)^48^, used the international IntCal20 database.

The existing historical records (e.g.,^25^) (the first eruption was reported in 1723) were reviewed and revised in order to complete the stratigraphy. From all those mentioned in the available historical records, only four of them are recognizable in the stratigraphic record, i.e., they produced deposits that can be identified in the field (1723–1724, 1917–1921, 1939–1940 and 1963–1965). Therefore, only those events represented by the larger tephra units were incorporated into the final stratigraphy of the last 2.6 ka of Irazú volcano.

To determine the ages of the tephra units, the paleosol samples were from the base and the top of the layer of interest, which allowed us to enclose the pyroclastic unit between two age ranges and to establish an intermediate age based on the ranges of variation of the paleosol ages. In those cases where a tephra unit was not dated, but there are ages in upper and lower tephra layers, an age was estimate based on the grade of paleosoil development (thickness, color, relative content of clay), based on the fact that environmental conditions have not changed significantly during the period under study. Therefore, the 18 ages by C14 for the last 2.6 ka allow us to assert that there is a good precision to assign ages to the studied units.

To evaluate the past eruptive frequency and estimate the possible occurrence of future eruptions we have followed the methodology proposed and applied by^49–51^. This methodology uses a cumulative event plot including each event according to its date of occurrence, thus highlighting the main quiescence periods and the main phases of activity during the period under study.

## Results

### Main characteristics of the Upper Holocene tephra deposits

**Figure 4:**
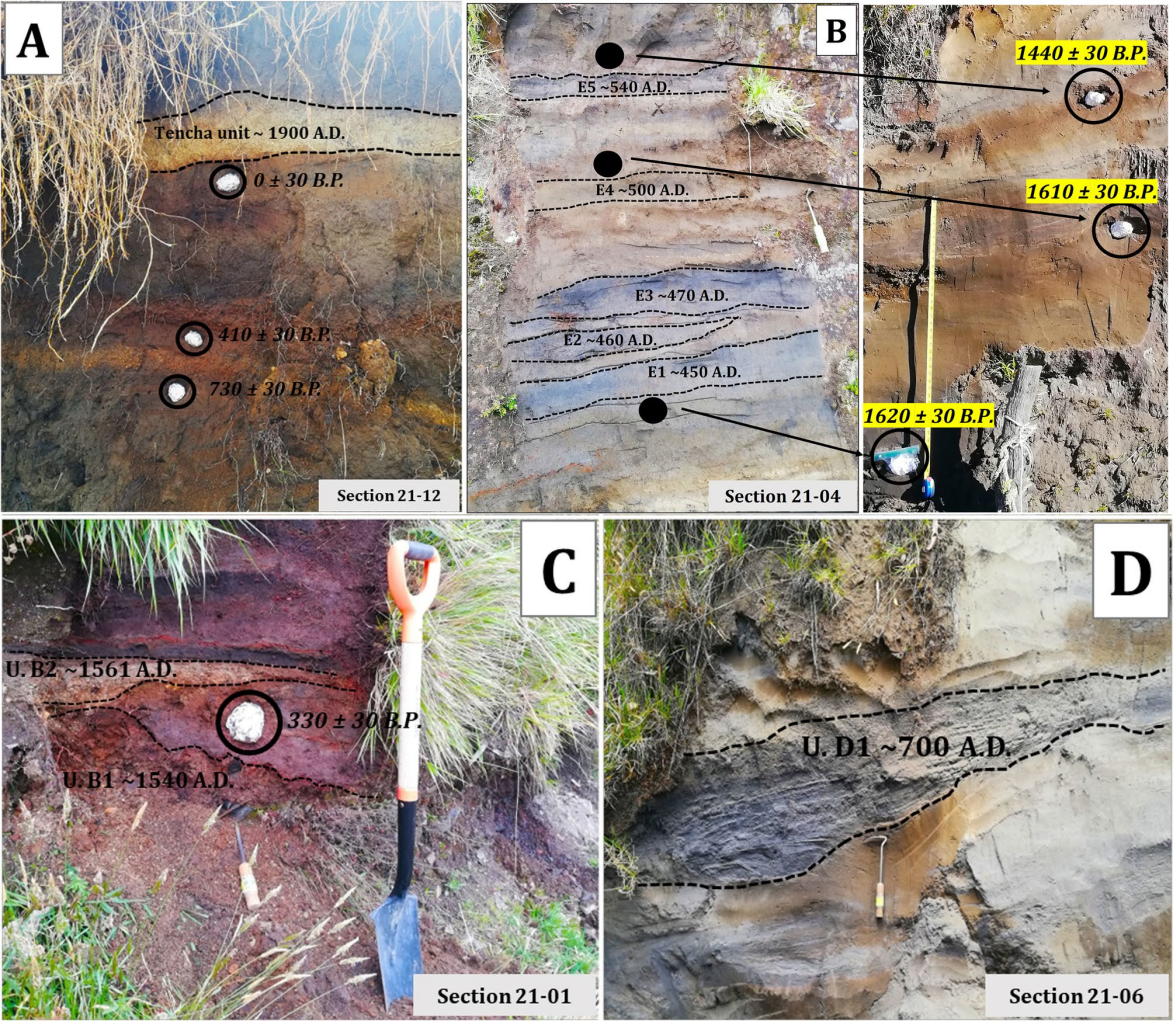
(**A**) A paleosol dated at 0 ± 30 yr B.P. easily recognizable in Units 21–12 and 21–13, both NE of the summit, under Tencha unit (avalanche deposit). The age indicates that this event would have occurred around 1900 A.D. The black circles indicate the points where paleosol samples were taken. (**B**) Units E1 until E5 represent one of the most important explosive episodes at Irazú from the last thousand years and are recognizable in the field, separated by thin paleosols. These units are restricted by two relatively close ages, 1620 ± 30 and 1440 ± 30 yr B.P. The black circles indicate the points where paleosol samples were taken. (**C**) Deposits associated with units B1 (~ 1540 A.D.) and B2 (~ 1561 A.D.), separated by a paleosol dated at 330 ± 30 yr B.P. (it is showed with a black circle) in the section 21–01. (**D**) Unit D1 (informally called the Shining layer in the field, due to its freshness) is recognized as resembling in color, texture, and grain size at the gray ash layers of the 1963–1965 eruption and is easily observed in sections 21–06, 21–07, and 21–08.

**Figure 5:**
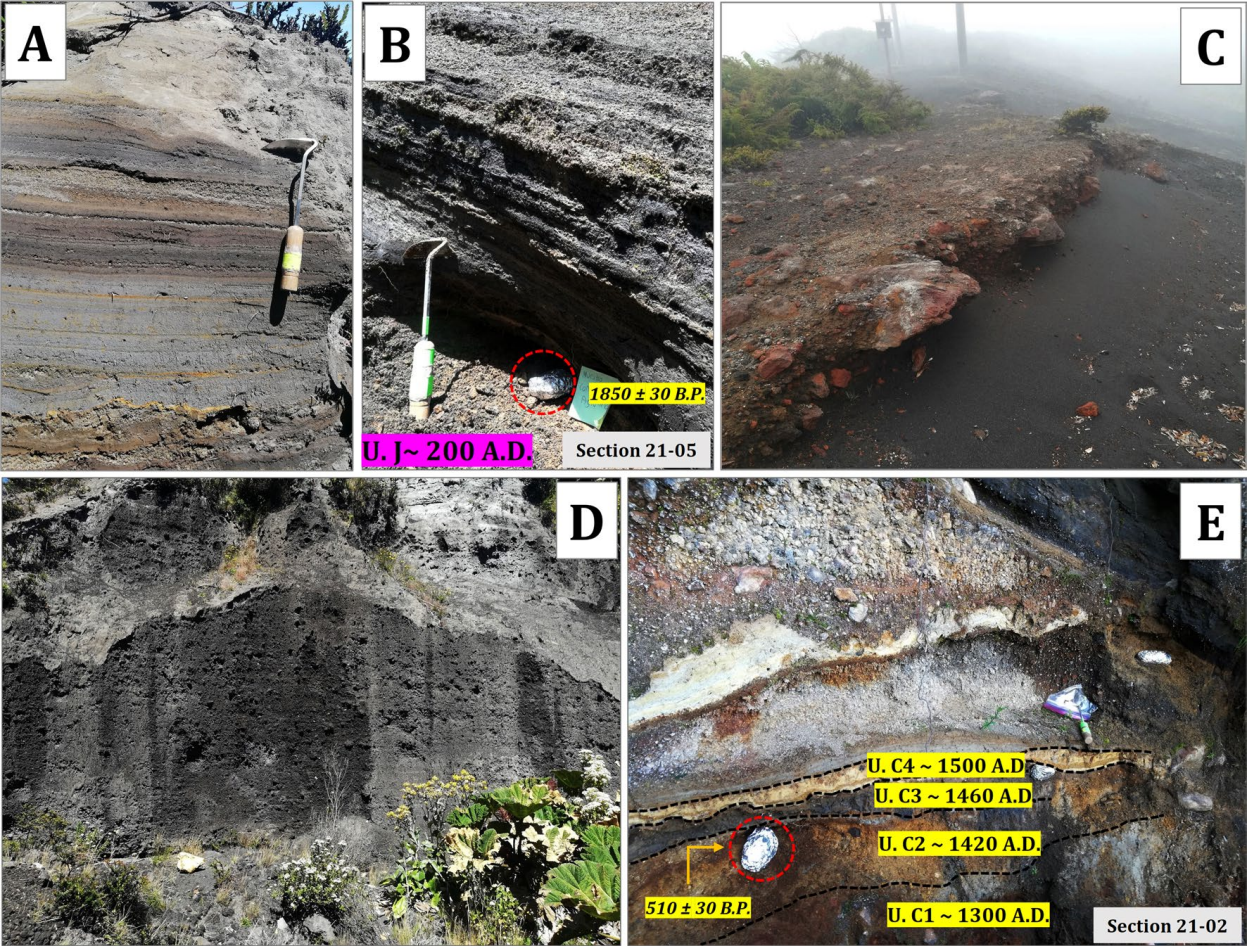
(**A**) Phreatomagmatic deposits associated with the 1963–1965 eruption. Deposits are located on the summit near section 21–14. (**B**). Paleosol dated at 1850 ± 30 yr B.P. on the agglutinate cropping out at the summit of Irazú (section 21–05). The estimated age of this Unit (J or Tristán) is ~ 200 A.D. The red circle indicates the point where paleosol sample was taken. (**C**). Agglomerate deposit (level of welded bombs), which outcrops throughout the Las Torres sector on the western summit of Irazú. (**D**) Fallout scoria deposit associated with the 1723 eruption, near the Main crater. E) Outcrop 21–02, where four units (C1 to C4), with ages chosen between 1300 and 1500 A.D, and a paleosol developed on unit C2 and dated at 510 ± 30 yr B.P., were identified. The red circle indicates the point where paleosol was sampled.

The lithological (grain size, composition of components, and their morphology and texture) and sedimentological (internal grading, stratification, thickness variations, and facies changes) description of the tephra deposits are summarized in Table 2. These characteristics were used to identify the nature of tephra layers (Table 4) (Figs. 4 and 5). They mostly correspond to fallout deposits, originated either by magmatic, phreatomagmatic, and/or phreatic processes, as can be deduced from the lithological and sedimentological characteristics of the deposits (Table 2). Their components mainly correspond to juvenile scoria fragments of basaltic and andesitic composition^19^ and of variable sizes (ash, lapilli, bombs) and lithic fragments (fragments of host rock) of older volcanic rocks, often hydrothermally altered, probably by post-eruptive fumarolic activity. The proportions of each component vary depending on the type of deposit (Table 2). The poor preservation of most of the studied tephra deposits and their high degree of weathering do not allow to establish isopach maps. However, variations in thickness of the deposits, as well as their spatial distributions and stratigraphic correlations were used to infer the source of some deposits.

**Figure 6:**
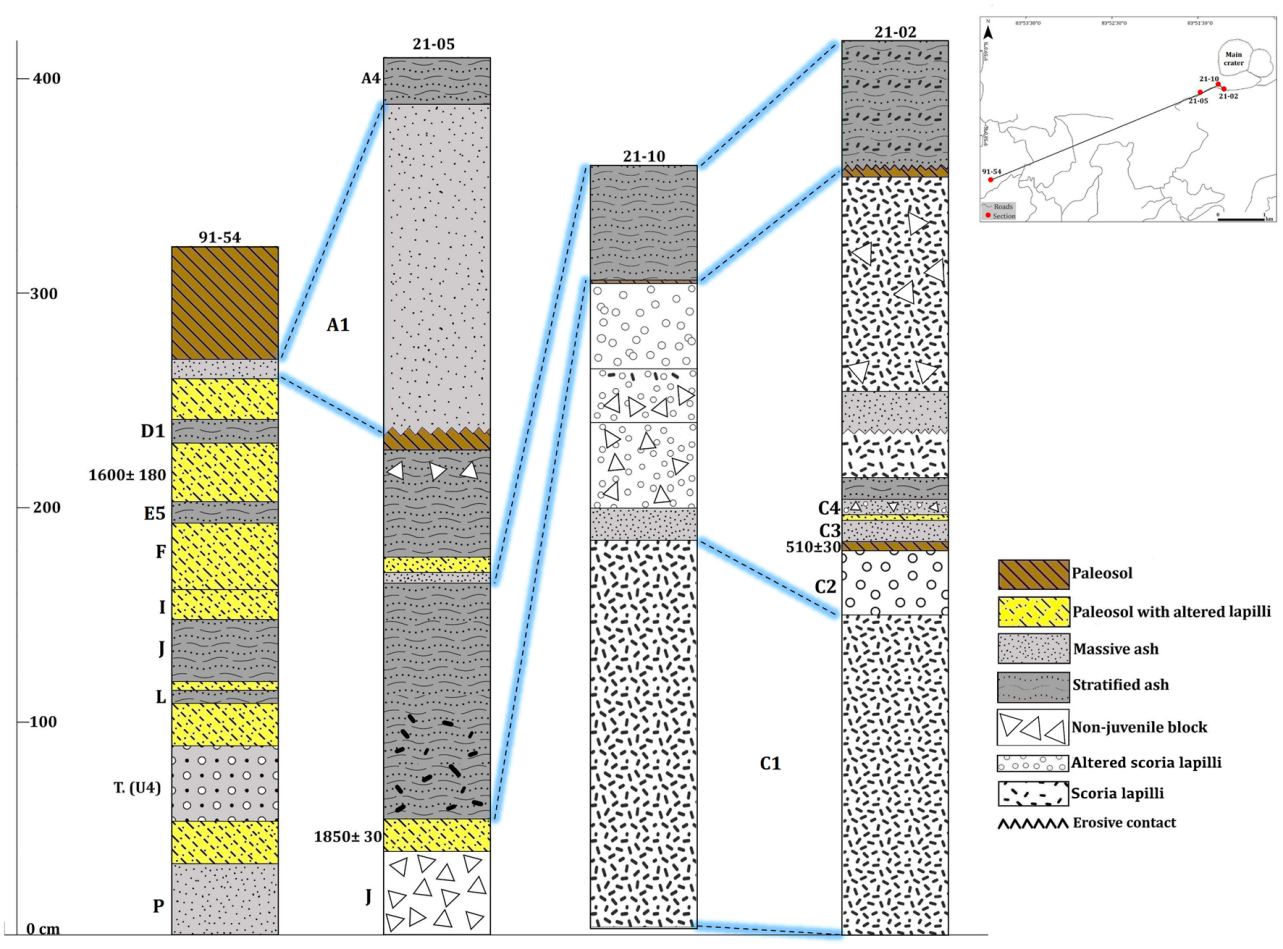
Stratigraphic correlation of the tephra deposits on the summit and SW and NE flanks of Irazú volcano. Plate 1 (see text for more explanation).

**Figure 7:**
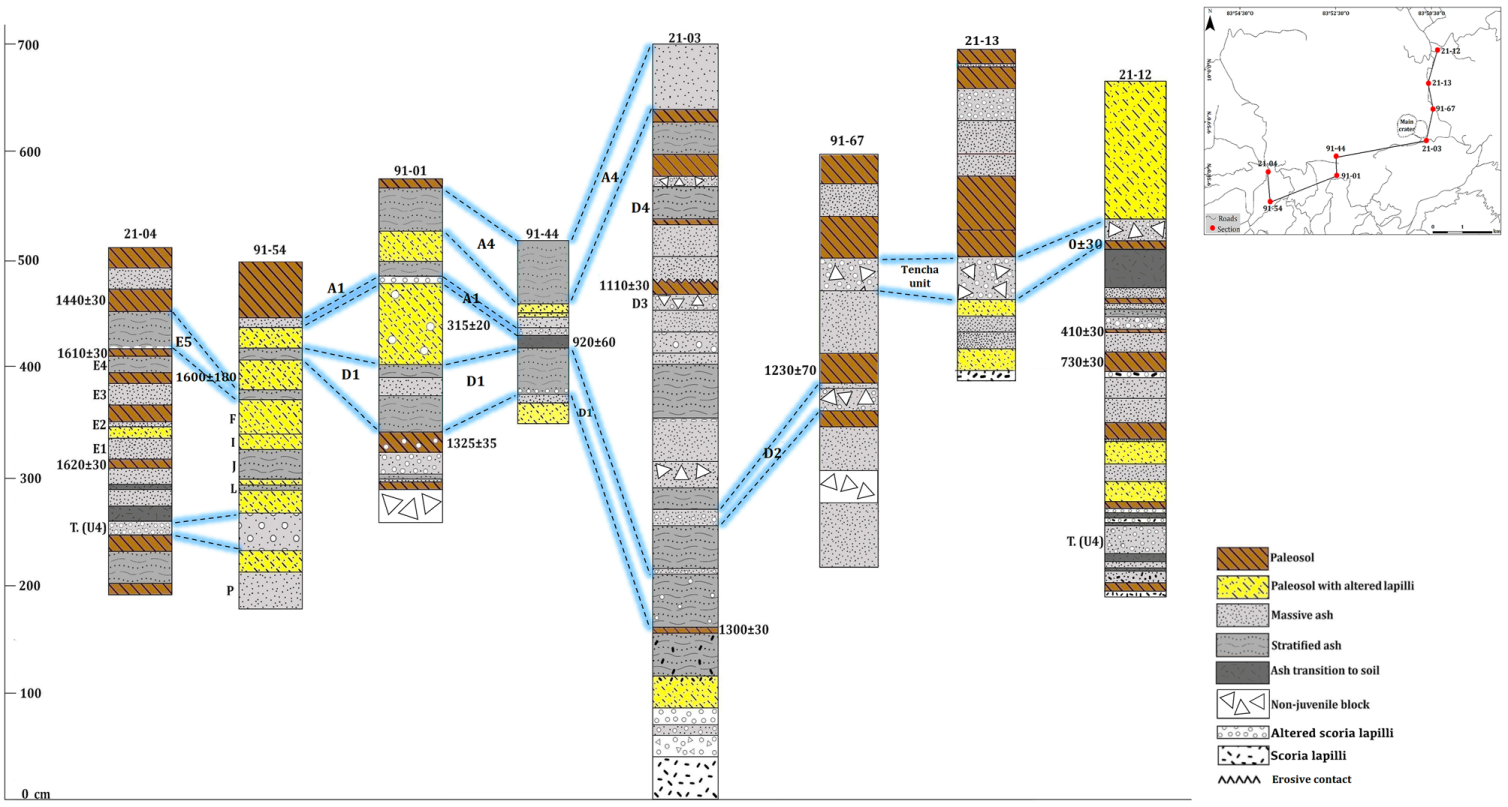
Stratigraphic correlation of the tephra deposits on the summit and SW and NE flanks of Irazú volcano. Plate 2 (see text for more explanation).

**Figure 8:**
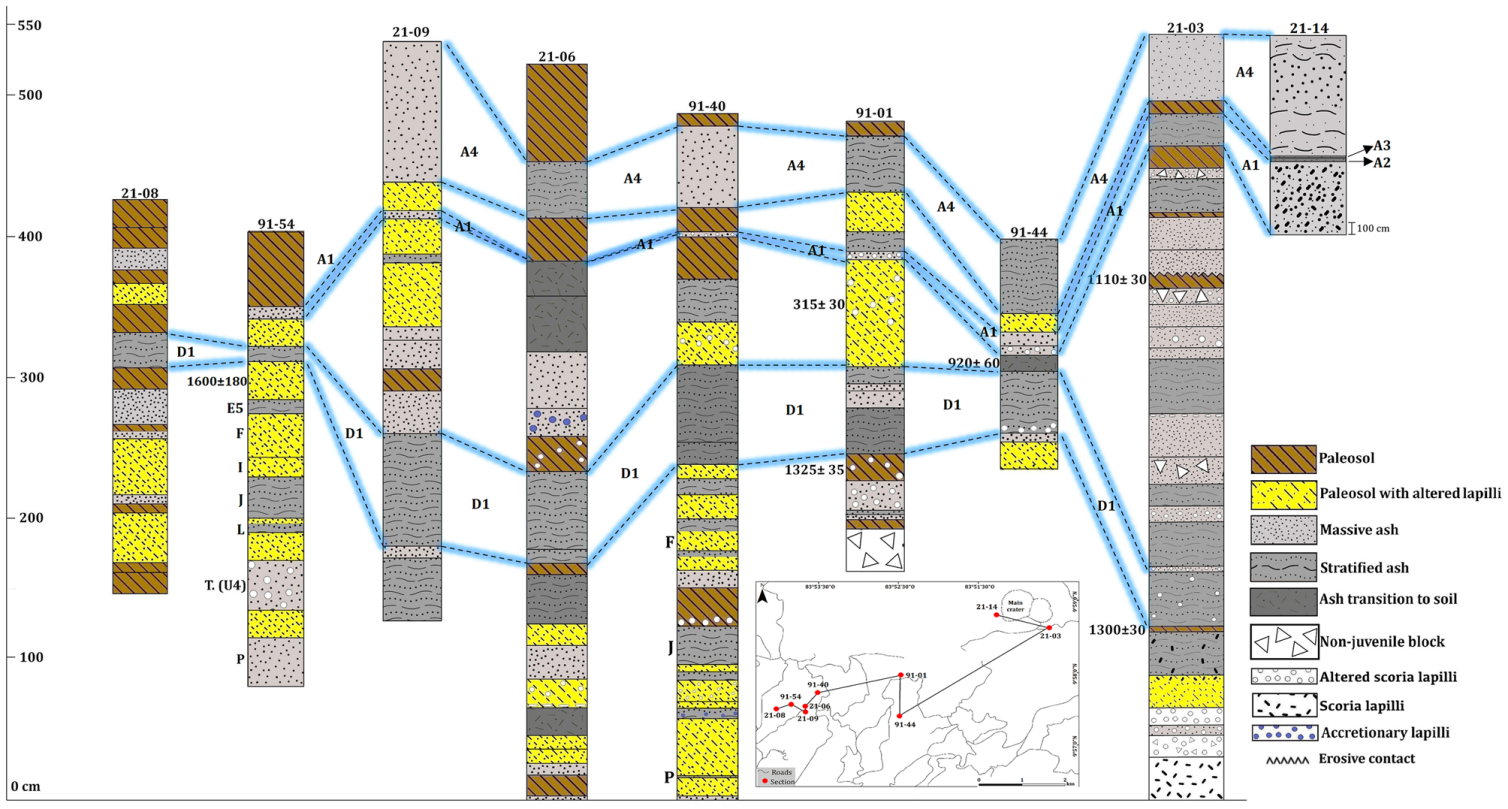
Stratigraphic correlation of the tephra deposits on the summit and SW and NE flanks of Irazú volcano. Plate 3 (see text for more explanation).

Most of these tephras are non-consolidated but locally they may appear partially lithified and endured. Fallout deposits may correspond to intermediate to distal (3- > 6 km) ashfall deposits (massive or stratified) (Fig. 4B,D), proximal to intermediate (< 0.5–2 km) stratified scoria lapilli deposits (Fig. 5B,D), or proximal (0- < 1 km) agglomerates and agglutinates deposits (Fig. 5C). Some deposits formed by accumulation of ballistic blocks, probably ejected during phreatic explosions, are also present (sections 21–05 and 91–01 in Fig. 6). The thickest (up to 2 m) fallout deposits (unit C1, sections 21–02 and 21–10 in Figs. 6 and Fig. 3 for location) are located at the summit and on the SW flank of the volcano (proximal area: < 1 km of the Main crater), which suggests a dominant wind towards that direction at the time of these eruptions, as no clear signs of erosion and/or remobilization are evident on this deposit. In addition to the large variety of fallout deposits, some small pyroclastic density current (PDC) deposits have also been identified, all corresponding to dilute PDCs, reaching a few kilometers from the main crater. These deposits are fine grained, well-stratified, sometimes thinly laminated or showing crossbedding, and containing mostly juvenile ash fragments with occasional scoria lapilli and lithic fragments of variable lithologies of the same sizes (Table 2). Thicknesses of the studied pyroclastic deposits may vary from one centimeter to several tens of meters in the proximal area (Figs. 6, 7, and 8). The grading can be normal, inverse, non-graded, or combinations of both. Fallout layers and dilute PDC deposits are usually well sorted, while agglomerates, agglutinates, and proximal explosion breccias are poorly-sorted (Figs. 4 and 5). Bioturbation is frequent (animal holes, trunks, and root effects), and remobilization structures (erosion, slumps, landslides) may be locally present. In addition, desiccation crack structures can be observed in the vicinity of the Main crater.

Contact relationships between the analyzed deposits can be (Figs. 4, 5 and 6): (a) conformable, with no erosion or significant hiatus between deposits; (b) erosive; or (c) unconformable, mainly corresponding to an angular unconformity between consecutive deposits, mainly in the proximal area. Near the summit and towards the Cabeza de Vaca, Retes, and Prusia hills (Fig. 3), local erosional unconformities and hiatuses between the pyroclastic sediments are frequent.

The presence of paleosols between pyroclastic units is common. The thickness of the paleosols can range from < 2 cm to > 6 m depending on the volcano flank and the distance from the source region (vent) of each deposit. The edaphological development varies from dark soils (rich in organic matter contents) to brown soils (with more edaphic development or with a certain degree of weathering).

### Tephrostratigraphic units of the Upper Holocene

The stratigraphic unit P (Figs. 3 and 8, sections 91–54 and 91–40), with a nearly constant thickness of 21 cm (distal area: 5 km SW from the Main crater), corresponds to gray ash, fine to coarse in size, with orange and pinkish levels with discontinuous wavy layers, and its age is ~ 900 B.C. being the oldest layer identified in the last 2.6 ka. Unit O (Fig. 3, section 91–53), with age of ~ 800 B.C. corresponds to a very fine gray ash deposit, and its thickness varies from 25 to 52 cm (distal area: 6 km SW from the Main crater). Units N, M and L (Figs. 3 and 6, 7, and 8, sections 91–32, 91–40 and 91–54), correspond to ash and lapilli rich deposits are underlain by the subplinian deposit of the Turrialba volcano (marker layer, T. U4), which can be identified in the whole study area (Figs. 6, 7, and 8, sections 9–54, 21–04, and 21–12). According with the C^14^ ages these eruptions occurred between ~ 300 and ~ 100 B.C. Unit K (Fig. 3, sections 91–46 and 91–47) is a thin, discontinuous ash layer (maximum thickness is 10 cm in the distal area: ~ 3 km SW from the Main crater and that towards the base presents discontinuous layers of brown to orange color; its age is ~ 100 A.D.

In the stratigraphic section 21–05 (Fig. 6) (see Fig. 3 for location), we identified an important layer with thicknesses between 1.0 and 1.4 m (proximal area: < 1 km of the Main crater), which corresponds to a scoriaceous agglutinate, locally showing features of a lava. It shows vertical fractures that resemble a poorly developed columnar jointing and seems to thin towards the Main crater. Radiocarbon dating on the paleosol overlying this layer provided an age of 1850 ± 30 yr B.P. (calibrated age 120–248 A.D.) (Figs. 5B and 6), so we have named this deposit unit J (Fig. 3, sections 21–05, 91–40 and Table 4) assigning to it an age of 200 A.D. This same unit was identified as the Tristán unit by^21^. At the site where the dating was obtained, the agglutinate has at least three levels of overlying paleosols, with intercalated tephras, culminating at the top with the historical tephra deposits of 1723–1724 and 1963–1965 (Figs. 6, 7 and 8). In other sectors where it corresponds to a stratified scoria lapilli deposit, which were subjected to streamflow and strong wind erosion, the 1723–1724 historical tephras directly overlie it (Fig. 6, section 21–05).

In stratigraphic section 21–04 (see Figs. 3 and 7) we dated three paleosols with organic matter (thicknesses between 5 and 20 cm) the paleosol under unit E1 gave an age of 1620 ± 30 yr B.P, the second paleosol (between units E4 and E5) was dated in 1610 ± 30 yrB.P., and finally, the paleosol above unit E5 gave an age of 1440 ± 30 yr B.P. (Figs. 4B and 7).

Unit E1 (lower) (Fig. 7, section 21–04) is a layer of dark gray, medium-grained ash interbedded with a soil (4 cm thick) overlain by a poorly defined brown paleosol (5 cm thick). This is followed by a level of gray ash, massive, medium to fine-grained, with a level of occasional light-color scoria lapilli fragments (6 cm thick), overlain by a paleosol rich in ash and fragments of organic matter (Unit E2). This is overlain by a poorly defined level of gray, massive, medium to fine-grained ash (10 cm thick, Unit E3), on which a poorly defined brown to gray soil developed, with occasional charcoal fragments (15 cm thick). Next, there are gray, fine, and massive ash layers (Units E4 and E5), with an intermediate hydrothermally altered level of ash and a thin paleosol in the middle (5 cm thick). According with the dating these units (from E1 to E5) have ages between ~ 450 and ~ 540 A.D., i.e., approximately 90 years of recurrent activity in time. These units are located in the distal area: ~ 5.5 km SW from the Main crater.

Unit D1 (Fig. 6b,c, sections 91–54, 91–01, 91–44, 21–03), corresponds to a stratified deposit rich in lapilli and coarse gray ash juvenile fragments; its thickness varies between 8 and 55 cm (maximum distance: ~ 5.5 km SW from the Main crater). It presents lateral thinning, an eroded upper surface with filling structures, and oxidized levels towards the top. It is of bright gray color, well stratified, and presents internal parallel and cross lamination and absence of cohesion (21–03, 21–06, 21–09, 21–08) (see Fig. 3 for location and Figs. 4d, 7 and 8). This unit is overlying a paleosol dated in 1300 ± 30 yr B.P. (Figs. 7 and 8, section 21–03, calibrated age 740–773 A.D.);^20^ obtained an age of 1325 ± 35 yr B.P. (section 91–01), similar to ours. Therefore, we can say that both ages are associated with unit D1. These calibrated ages provide maximum ages between 740–773 and 657–687 A.D., respectively (Table 4). Therefore, 700 A.D. would be the most appropriate age for this relevant event.

Unit D2 (Figs. 7 and 8, section 21–03) (~ 20 cm thick) corresponds to a pyroclastic deposit rich in lapilli and intermediate ash size fragments of white and orange colors. It lies below a paleosol dated to 1230 ± 70 yr B.P. (780–883 A.D.). Unit D3 (Figs. 7 and 8, section 21–03), (45 and 55 cm thickness) corresponds to a gray to brown ash deposit, sometimes massive, with the presence of lapilli size fragments and hydrothermally altered blocks. It is underlain by a paleosol dated 1110 ± 30 yr B.P. (895–990 A.D.), so an age of ~ 850 A.D. have been assigned to it. Overlying this paleosol is Unit D4 (Figs. 7 and 8, section 21–03), composed of fine to very fine ash; its base (first 14 cm) is massive and has pink horizons, while the middle part (15 cm) is stratified and towards the top is deformed (9–23 cm thick). It is followed by a level rich in orange to white scoria lapilli fragments, poorly sorted in a red clay matrix (15–29 cm thick). According to ages of the associated paleosols eruptions responsible for deposits D2, D3, and D4 probably occurred around an age range between 800 and 1000 AD (Table 4). These units are located in the proximal area: < 1 km SE from de Main crater).

Unit C1 (Fig. 6, sections 21–10, 21–02), appears in the proximal area (< 1 km NW from de Main crater) and corresponds to a deposit of coarse-grained black ash to vesicular scoria lapilli clasts. In the proximal area it can be correlated with a 1.8 m thick deposit of coarse scoria lapilli to bomb (sections 21–02 and 21–10) (see Fig. 3 for location and Figs. 5E and 6). Unit C2 (Fig. 6, section 21–02) (20–40 cm thick) is composed of a massive and poorly sorted hydrothermally altered deposit rich in lapilli fragments (orange, brown, gray), locally with block impacts (Fig. 5E). It is overlain by a discontinuous brown to orange paleosol with a maximum thickness of 4 cm (section 21- 02), which is dated to 510 ± 30 yr B.P. (calibrated age 1409–1434 A.D.). Comparing the age of this paleosol with the other ages we may assume that Units C1 and C2 were deposited probably around 1300 A.D. and ~ 1420 A.D. Using the thickness of the deposits in the proximal area, we can estimate that the eruptive focus of both events was located at the summit of the Irazú volcano (near the Main crater).

On top of Unit C2, there is a level of gray ash, with perturbation (physical and biological) and lateral wedging (10 cm thick); we have named this as Unit C3 (Fig. 6, section 21–02) (age ~ 1460 A.D.) (Fig. 5E and Table 4). At the top, there is an erosional unconformity and a paleosol with hydrothermally altered tephras. Unit C4 (Fig. 6, section 21–02) (7 cm thick) is a phreatic ash level (brown to gray), with lapilli size fragments and non-juvenile blocks and desiccation cracks in the fine ash, with an age of ~ 1500 A.D. It may present black scoria, although hydrothermally altered fragments predominate. An erosional unconformity defines the top of the succession (Fig. 5E).

**Figure 9:**
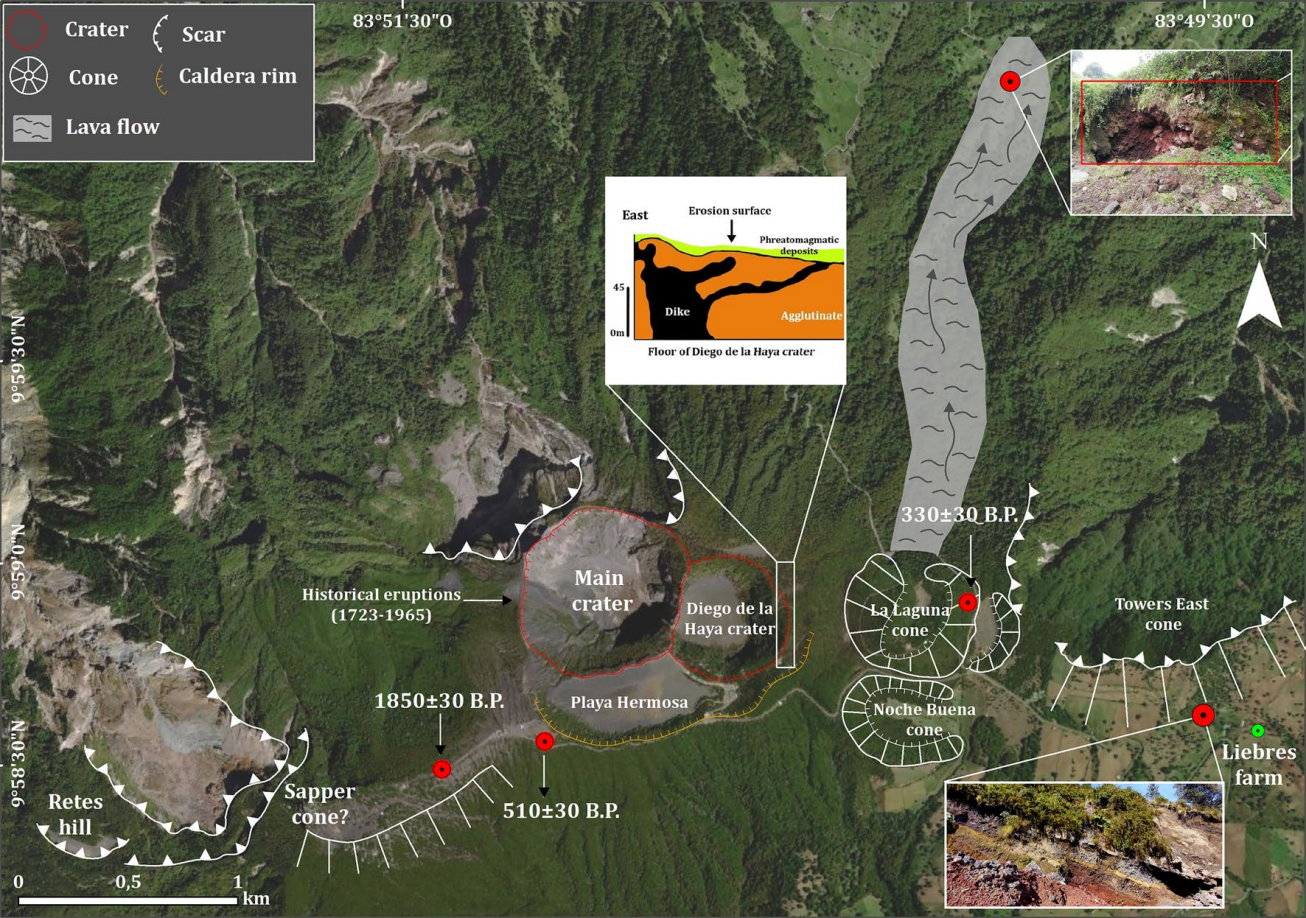
Detail of the E-W fissure, showing the geoforms (craters and pyroclastic cones) and their associated ages. To the north of the pyroclastic cone La Laguna is probably the most recent lava flow identified in this investigation and at the east end is the avalanche scar of the pyroclastic cone East Towers (so called in this investigation).

We dated a paleosol on a scoria layer (≥ 1 m thick) associated with the La Laguna cone (stratigraphic column 21-01) (intermediate area: ~ 1.5 km E from de Main crater) (see Fig. 3 for location and Fig. 4C). The radiocarbon age provided was of 330 ± 30 yr B.P. (1549–1598 A.D.), which is very close to that cited by Clark^20^ in 315 ± 20 yr B.P. (1521–1577 A.D.). This paleosol separates two tephra levels B1 and B2 (Fig. 3, section 21–01) (Fig. 4C). Unit B1 (~ 1540 A.D.) corresponds a deposit composed of scoria lapilli. In addition, near the summit of Irazú, there are stratified ash and lapilli scoria in the same deposit (55–100 cm thick) underlain by fine-grained layers, perhaps contemporaneous with the La Laguna cone. We also identified a lava flow (3 km long) to the N of La Laguna cone, which could be contemporaneous with the formation of this cone and (section 21–01), therefore, it could be one of the most recent lava flows in Irazú (≤ 1500 A.D.) (Fig. 9). Unit B2 (~ 1561 A.D.) contains lapilli and ash size juvenile fragments with abundant lithic fragments of the same sizes and of different lithologies.

A paleosol dated in section 21–12 (Fig. 3 for location and Figs. 4A and 7) with an age of 0 ± 30 yr B.P. (calibrated age 1876—1916 A.D.) is located under a distinctive deposit with strong thickness variation (between 20 and 40 cm) even in the same outcrop (distal area: 3 km NE from the Main crater). This layer shows badly sorted white and orange angular clasts of various sizes and different degree of hydrothermal alteration, in an altered fine matrix. It is easily recognizable to the NE of the Main crater (on the road to San Gerardo Dairy), especially along the road cuts (sections 21–12 and 21–14) (Fig. 7) near a landslide scarp associated with the Toro Amarillo River canyon (Fig. 2 to location). This deposit is not characteristic of a primary volcanic tephra. By comparison with similar deposits generated by sector collapses at the N flank of Irazú in historical and prehistorical times, which show hummocky surfaces, jigsaw-fit clasts and facies mixing soil injections^52^, this deposit resembles more an overbank avalanche or landslide deposit. This non-volcanic unit is an important guide layer for the stratigraphy at the NE sector, and for this reason we named it Tencha unit (Fig. 7, sections 21–12 and 21–13) (~ 1900 A.D.) (Fig. 4A), which was identified^20^ as unit Q in his study.

Regarding historical tephras, for the period comprised between 1723 and 1917, and despite there are some mentions in the written records to possible activity events at Irazú, the bibliographic review we conducted, as well as the field inspection, did not find historical and stratigraphic evidence to support them. We assumed that most of these supposed eruptive events were confused with fumarolic activity or strong earthquakes. A similar conclusion was reached by^25^ and^19^. For this reason, only those events represented by specific tephra units, this is those corresponding to 1723–1724, 1917–1921, 1939–1940 and 1963–1965, were incorporated into the final stratigraphy. The deposits from the 1723–1724 and 1963–1965 eruptions have been extensively studied, perhaps the best and most reviewed pyroclastic deposits in Costa Rica^19,22,53–57^. Unit A1 (Figs. 7 and 8, sections 21–05, 91–54, 91–01, 91–44, 21–03) (tephra from the 1723–1724 eruption) corresponds to black basaltic andesitic scoria with occasional andesitic white pumice^19^ and phreatomagmatic ash layers and thick phreatomagmatic breccia deposits that are mainly recognizable at the summit of Irazú (Figs. 5D, 7 and 8). The tephras from the 1917–1921 (Unit A2) and 1939–1940 (Unit A3) eruptions, are limited to the summit (Fig. 3 section 21–14 and Fig. 6C) and correspond to thin levels (0.5 to 4 cm thick) of ash fallout and dilute PDC deposits, with yellow to red coloration, being separated by local erosional unconformities^19^. Finally, unit A4 (Figs. 7 and 8, sections 91–01, 91–44, 21–03, 21–14, 21–09, 21–06, 91–40) (tephra from the 1963–1965 eruption) includes different deposits of dense and dilute ash-rich PDC deposits and ash-fall layers, as well as phreatomagmatic and Strombolian deposits and rare thin (few centimeters), fine ash phreatic deposits (Fig. 5A). All the historical eruptive events had their origin in the Main crater and all these units have a distinctive geochemical composition^21^.

## Discussion

### Vents location

Despite this study does not present any isopachs map that could contribute to track the dispersal of the Irazú tephras, some characteristics of these deposits provide insights to infer the possible location of their sources. Tephra deposits corresponding to the units P to A4 (Table 4) appear in sections that are located about 6 km SW and 3 km NE of summit (see Fig. 3). The recognizable volcanic morphologies (pyroclastic cones and craters), the existence of proximal coarse tephra deposits (deposits of bombs, agglutinates, and agglomerates), and their areal distribution and thickness variations in the columns and outcrops (see Figs. 3 and 6, 7, and 8), as well as the preferential emplacement directions followed by the corresponding deposits, suggest the existence of a E-W fissure at the summit of Irazú on which different eruptive foci were active over the last 2.6 ka (Fig. 9). This fissure was already suggested by^58^ who indicated that craters at the summit of Irazú were aligned from east to west striking N80°W. Moreover, Alvarado^19^ suggested that this alignment of cones and craters corresponded to a migration of the eruptive focus in an E-W direction, lying the oldest cone to the east, near Finca Liebres (Fig. 9) and becoming successively younger towards the west.

In this research we discuss for the first time the evolution of this eruptive fissure and its associated craters and cones. If we analyze the ages of these proximal deposits, it is interesting to observe that the eruptive activity in this fissure have not presented a specific pattern in the last 2.6 years (Fig. 9 and Table 4). The eruptive foci (called Sapper) of the oldest eruptions, from 1000 A.D., i.e., units P to D4, would have been located near the present position of the Sapper hill (W of the fissure), as suggested by the areal extent toward the SW flank and thickness variations of the deposits, which widen and get thinner, respectively, from that point. Alvarado et al.^21^ proposed that the Tristán Unit (Unit J in Fig. 6, sections 21–05, 91–40) may indicate that the source was located adjacent to the Sapper hill at the summit of Irazú (Fig. 9).

Unit C1 allows to estimate another possible eruptive focus due to the fact that, near the summit, this deposit presents a thickness greater than 1.8 m and is composed of coarse lapilli scoria and bombs (sections 21–02 and 21–10) (Fig. 5E). Consequently, we can estimate that the vent of this tephra layer was located near the summit. The paleosol dated on this deposit (510 ± 30 yr B.P.) permits us to place this event at ~ 1300 A.D. (Table 2).

Unit B2 is located above a paleosol in La Laguna cone at E of the fissure (Fig. 4C) dated at 330 ± 30 yr B.P. (1549–1598 A.D.). This event could be the origin of the lahars mentioned in the legend of Irazú associated with a possible eruption of ~ 1561 A.D. Alvarado et al. and Horn^21,59^ dated a paleosol to 315 ± 20 yr B.P. (1521–1577 A.D.) and related it to the event recorded in 1561; however, both ages and the stratigraphic record (Sections 21–01) indicate that these are two different events (B1 and B2 Units) separated by thin paleosols, but which occurred very close in time (B1: ~ 1540 A.D., and B2: ~ 1561 A.D.) (Fig. 4C). In addition, near the summit of Irazú, there are a stratified level of ash and lapilli fallout scoria levels (55–100 cm thick) underlain by layers only composed of lithic fragments (phreatic), and layers including juvenile and lithic components (phreatomagmatic), perhaps contemporaneous with the La Laguna cone. It is possible that several contemporaneous active vents coexisted. In such a case, the La Laguna cone was formed around 1540 A.D., while the overlying tephra level was from 1561. A prehistoric scoria cone and two tuff rings are located further to the east. Another composite crater is represented by prominent cliffs immediately to the south of the Main crater and Diego de La Haya vents (Fig. 9)^21^. Alvarado et al.^21^ also identified a phreatic/phreatomagmatic breccia (Alfaro unit) with a thickness of ~ 4 m at the SE of Main Crater with a stratigraphic position pre-1723. This would suggest that these units correspond to the same event, which originated the Main crater around XVI or XVII centuries, i.e., before the eruption of 1723, the first historical eruption registered at Irazú. Therefore, this crater would be recent, and its formation would correspond to one of the most important phreatomagmatic events at Irazú over the last 2 ka.

In historical times, Elizondo et al.^40^ point out that the first documentary record indicates that between 1899 and 1916, at least 12 small vents (intracrater foci) were present in the sector where the current Main Crater is now located, which merged in subsequent eruptive periods (e.g., 1917–1921, 1924,1928, 1930, 1939–1940 and 1963–1965) to form the current Main Crater. All this evidence allows us to conclude that the E-W fissure has been active and presents an important geomorphologic evolution for both prehistoric and historic times (Fig. 9).

### Frequency and eruptive styles

The tephrostratigraphy and tephrochronology of Irazú volcano presented in this study reveal that it has not shown a uniform eruption frequency during the last 2600 years. Due the detailed tephrostratigraphy analyzed in this study and the incompleteness of the older stratigraphic record, we suggest using this most recent period to evaluate the potential of future eruptions at Irazú volcano.

**Figure 10:**
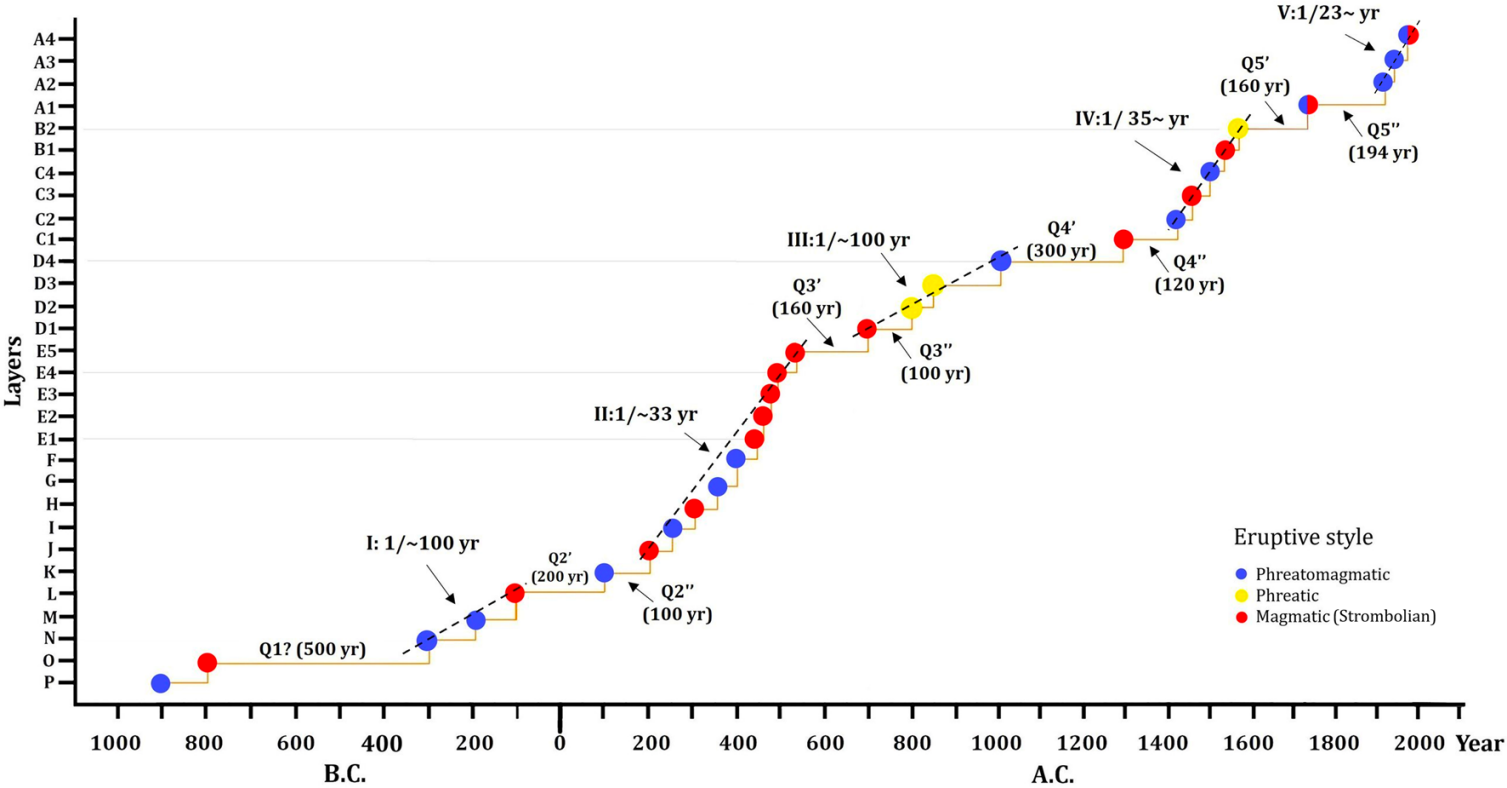
Eruptive frequency of the main units given in the literature and the new data provided in the present study, where different rates of activity are observed. The eruptive frequency has been higher in the last 2.6 ka due to improved chronostratigraphic sampling. Some quiescent periods are also distinguished. Also, the eruptive styles of the eruptions registered in the last 2.6 ka are showed.

Considering together the prehistoric data analyzed in this study and the available historical records, this provides an average of at least one eruption every 85 years. Moreover, the new data allow us to identify some important phases and quiescent periods where no activity has been registered (Fig. 10). From the data obtained, we have developed a diagram of cumulative eruptive frequency for the last 2.6 ka, in which at least five main eruptive periods—with different phases in each and separated by a quiescent period—can be established (Fig. 10). The first eruptive period (I), with a recurrence of one eruption approximately every 100 years, was preceded by a period without an eruption (Q1) of apparently 500 years. This eruptive period was followed by a ~ 200 years quiescent period (Q2’), which was interrupted by an eruption, followed by 100 years of further quiescence (Q2’’).

Probably due to better stratigraphic details and radiocarbon sampling, the second eruptive period (II) shows a higher eruptive frequency, with a recurrence of one eruption approximately every 33 years. In this sense, paleoclimatic studies in the sediments of Lago Chirripó, suggest intervals of lower lake level at about 1100 and 2500 yr BP^57^ that may be associated with regional droughts in the Holocene^60,61^, which could explain the preservation of tephras for this period.

This period was followed by a quiescent period of 160 years (Q3’), which was interrupted by an eruption (Unit D1) that was succeeded by 100 years of quiescence (Q3’’). We identify a third eruptive period (III), which is represented by an event approximately every 100 years, followed by an important quiescent period of 300 years (Q4’). During this eruptive period,^62^ presented evidence of a prolonged period of low lake levels at Laguna Zoncho in the southern Pacific region of Costa Rica between 1220 and 840 yr B.P. (730–1110 A.D.), possibly indicating the influence of the Terminal Classic Drought (TCD) in southern Costa Rica. Also, Horn^59^ pointed out that evidence of drying in Central America suggests that severe droughts between 1300 and 1100 yr B.P. may have been common and widespread. Therefore, these climatic conditions may have favored the preservation of the tephra deposits of the units present in this eruptive period.Between the Q4′ (300 years of quiescence) and Q4’’ (120 years of quiescence), we identified only one eruptive event (Unit C1). This last period without an eruption was interrupted by the fourth eruptive period (IV), which was characterized by one eruption every ~ 35 years. Subsequently, we have an important hiatus of approximately 160 years (Q5’) that was succeeded by one important eruptive event (Unit A1). After these eruptive episodes, we have identified a final period without an eruption of 194 years (Q5’’) that was followed by the fifth eruptive period (V), which registered one eruption every 23 years on average. It corresponds with the eruptions registered in historical time. The most important periods without eruptions are separated by only one event, which give rise to substantial eruptive events over time (Fig. 10). For example, periods Q3'–Q3″ and Q4'–Q4″ separated by significant eruptions such as those represented in Units D1 and C1, respectively.

Concerning the nature of volcanic eruptions, the lithological and sedimentological characteristics of tephra deposits provide the clues to identify corresponding eruption styles (Tables 2 and 4). In this sense, we have identified 30 tephrostratigraphic units in the last 2.6 ka, where 11 of them correspond to phreatomagmatic eruptions, 13 to magmatic (Strombolian) eruptions, two combined (phreatomagmatic and magmatic) and four phreatic (Fig. 10). It is notable the lack of lava flows during this period. Alvarado et al.^19^ indicated that many eruptive episodes of the Holocene at the summit of Irazú began with magmatic or “dry” eruptions and culminated with phreatic and phreatomagmatic eruptions, such as the case of the 1963–1965 eruption. Consequently, if the eruptive pattern and frequency are maintained, Irazú should be considered an active volcano that may erupt again in a few years or tens of years.

## Conclusions

New radiocarbon ages and an updated stratigraphy of Irazú’s summit and its SW and NE flanks have allowed us to define at least 30 major tephrostratigraphic units for the last 2.6 ka and to update the eruptive recurrence for this period. According to its geomorphological, stratigraphical, and radiometric ages, we suggest that the La Laguna cone and Main Crater formed in recent times (between ~ 1500 and ~ 1600 A.D.) and that Sapper hill was the source area of eruptions older than ~ 200 A.D. Therefore, volcanism has moved along a fissure zone with a E-W direction in the uppermost part of the volcano, which has been active during the Upper Holocene. Before this research, there was some debate about possible eruptions in the nineteenth century, however, none of the radiocarbon ages had dated any eruptive event close to this period, and the review of historical documents and field records does not support the occurrence of any eruption between 1724 and 1917. These results are supported by historical data indicating a quiescent period between 1723–1724 and 1917 (193 years). Therefore, our results confirm the rigor of our data and their interpretations. The data presented shows that Irazú volcano has significant eruptions between every 23 and 100 years. The volcano is currently in a state of potential re-activation, but its activity could increase progressively trending to a new eruptive stage in the next few years or tens of years. Therefore, and although our estimates of recurrence (based on stratigraphic records) may seem somewhat crude from a statistical point of view, they will be essential for conducting further studies of volcanic hazard and vulnerability analysis for the next eruptive period of Irazú. This is particularly relevant for the areas surrounding the volcano, and in the Reventado river basin, where there are highly-vulnerable population centers which may be severely affected by volcanic hazards (e.g., air fall, lahars).

## Data Availability

All data generated or analysed during this study are included in this published article, which does not contain any supplementary information file.
